# Correction: Efficient biomass saccharification using a novel cellobiohydrolase from *Clostridium clariflavum* for utilization in biofuel industry

**DOI:** 10.1039/d1ra90090k

**Published:** 2021-03-18

**Authors:** Asma Zafar, Muhammad Nauman Aftab, Anam Asif, Ahmet Karadag, Liangcai Peng, Hasan Ufak Celebioglu, Muhammad Sohail Afzal, Attia Hamid, Irfana Iqbal

**Affiliations:** Faculty of Life Sciences, University of Central Punjab Lahore Pakistan; Institute of Industrial Biotechnology, GC University Lahore Pakistan nauman535@yahoo.com +92-3444704190; Department of Chemistry, Faculty of Arts and Sciences, Yozgat Bozok University Yozgat 66200 Turkey; Biomass and Bioenergy Research Center, Huazhong Agriculture University Wuhan China; Department of Biotechnology, Bartin University Bartin Turkey; Department of Life Sciences, School of Science, University of Management and Technology (UMT) Lahore Pakistan; Department of Zoology, Lahore College for Women University Lahore Pakistan

## Abstract

Correction for ‘Efficient biomass saccharification using a novel cellobiohydrolase from *Clostridium clariflavum* for utilization in biofuel industry’ by Asma Zafar *et al.*, *RSC Adv.*, 2021, **11**, 9246–9261, DOI: 10.1039/D1RA00545F.

The authors regret that in the original article, the name and affiliation for one of the co-authors (Hasan Ufak Celebioglu) were incorrectly given. The correct name and affiliation are shown here.

The Royal Society of Chemistry apologises for these errors and any consequent inconvenience to authors and readers.

## Supplementary Material

